# Supplementary material for: FG-Droid: Grouping based feature size reduction for Android malware detection
Source: PeerJ Comput Sci. 2022 Jul 14;8:e1043. doi: 10.7717/peerj-cs.1043 (PMC9299264; doi:10.7717/peerj-cs.1043)
Supplement: Appendix A [file peerj-cs-08-1043-s003.docx]

| Feature Group | Included Features | Feature Group Details |
| --- | --- | --- |
| 1- Access | ACCESS_ALL_DOWNLOADS;ACCESS_BLUETOOTH_SHARE;ACCESS_CACHE_FILESYSTEM;ACCESS_CHECKIN_PROPERTIES;ACCESS_CONTENT_PROVIDERS_EXTERNALLY;ACCESS_DOWNLOAD_MANAGER;ACCESS_DOWNLOAD_MANAGER_ADVANCED;ACCESS_DRM_CERTIFICATES;ACCESS_EPHEMERAL_APPS;ACCESS_FM_RADIO;ACCESS_INPUT_FLINGER;ACCESS_KEYGUARD_SECURE_STORAGE;ACCESS_LOCATION_EXTRA_COMMANDS;ACCESS_MOCK_LOCATION;ACCESS_MTP;ACCESS_NETWORK_CONDITIONS;ACCESS_NETWORK_STATE;ACCESS_NOTIFICATIONS;ACCESS_NOTIFICATION_POLICY;ACCESS_PDB_STATE;ACCESS_SURFACE_FLINGER;ACCESS_VOICE_INTERACTION_SERVICE;ACCESS_VR_MANAGER;ACCESS_WIFI_STATE;ACCESS_WIMAX_STATE | It is the group that contains the permissions used to ACCESS various information such as downloads, Bluetooth, location information, internet network usage information, wi-fi status. |
| 2- Modify | MODIFY_APP;MODIFY_AUDIO_ROUTING;MODIFY_AUDIO_SETTINGS;MODIFY_CELL_BROADCASTS;MODIFY_DAY_NIGHT_MODE;MODIFY_NETWORK_ACCOUNTING;MODIFY_PARENTAL_CONTROLS;MODIFY_PHONE_STATE | The group contains the permissions used to MODIFY settings such as sound settings, access status, and network account information. |
| 3- Set | SET_ACTIVITY_WATCHER;SET_ALWAYS_FINISH;SET_ANIMATION_SCALE;SET_DEBUG_APP;SET_INPUT_CALIBRATION;SET_KEYBOARD_LAYOUT;SET_ORIENTATION;SET_POINTER_SPEED;SET_PREFERRED_APPLICATIONS;SET_PROCESS_LIMIT;SET_SCREEN_COMPATIBILITY;SET_TIME;SET_TIME_ZONE;SET_WALLPAPER;SET_WALLPAPER_COMPONENT;SET_WALLPAPER_HINTS | The group contains the permissions used to SET information such as activity monitoring, checking whether the activities are finished when the applications are running in the background, and animation scaling. |
| 4- Update | UPDATE_APP_OPS_STATS;UPDATE_CONFIG;UPDATE_DEVICE_STATS;UPDATE_LOCK;UPDATE_LOCK_TASK_PACKAGES | The group contains the permissions used to UPDATE configurations, information such as device statistics. |
| 5- Write | WRITE_CALENDAR;WRITE_CONTACTS;WRITE_EXTERNAL_STORAGE;WRITE_HISTORY_BOOKMARKS; WRITE_CALL_LOG | The group contains the permissions used for WRITING calendar information, contacts, storage information. |
| 6- Read | READ_CALL_LOG; READ_CALENDAR; READ_CONTACTS; READ_EXTERNAL_STORAGE;READ_HISTORY_BOOKMARKS;READ_PHONE_STATE;READ_SMS | The group has permissions used to READ calendar information, contacts, storage information. |
| 7- Get | GET_ACCOUNTS; GET_ACCOUNTS_PRIVILEGED; GET_APP_GRANTED_URI_PERMISSIONS;GET_APP_OPS_STATS;GET_DETAILED_TASKS;GET_INTENT_SENDER_INTENT;GET_PACKAGE_IMPORTANCE;GET_PACKAGE_SIZE;GET_PASSWORD;GET_PROCESS_STATE_AND_OOM_SCORE;GET_TASKS;GET_TOP_ACTIVITY_INFO | The group contains the permissions used to GET account information, authorization status of accounts, and employee tasks. |
| 8- Manage | MANAGE_ACCOUNTS;MANAGE_ACTIVITY_STACKS;MANAGE_APP_OPS_RESTRICTIONS;MANAGE_APP_TOKENS;MANAGE_CA_CERTIFICATES;MANAGE_DEVICE_ADMINS;MANAGE_DOCUMENTS;MANAGE_FINGERPRINT;MANAGE_MEDIA_PROJECTION;MANAGE_NETWORK_POLICY;MANAGE_NOTIFICATIONS;MANAGE_PROFILE_AND_DEVICE_OWNERS;MANAGE_SOUND_TRIGGER;MANAGE_USB;MANAGE_USERS;MANAGE_VOICE_KEYPHRASES | The group contains the permissions required for the MANAGEMENT of account information, authorization status of the accounts, and access to running tasks. |
| 9- Bind | BIND_ACCESSIBILITY_SERVICE;BIND_APPWIDGET;BIND_CARRIER_MESSAGING_SERVICE;BIND_CARRIER_SERVICES;BIND_CHOOSER_TARGET_SERVICE;BIND_CONDITION_PROVIDER_SERVICE;BIND_CONNECTION_SERVICE;BIND_DEVICE_ADMIN;BIND_DIRECTORY_SEARCH;BIND_DREAM_SERVICE;BIND_INCALL_SERVICE;BIND_INPUT_METHOD;BIND_INTENT_FILTER_VERIFIER;BIND_JOB_SERVICE;BIND_KEYGUARD_APPWIDGET;BIND_MIDI_DEVICE_SERVICE;BIND_NFC_SERVICE;BIND_NOTIFICATION_LISTENER_SERVICE;BIND_NOTIFICATION_RANKER_SERVICE;BIND_PACKAGE_VERIFIER;BIND_PRINT_RECOMMENDATION_SERVICE;BIND_PRINT_SERVICE;BIND_PRINT_SPOOLER_SERVICE;BIND_QUICK_SETTINGS_TILE;BIND_REMOTEVIEWS;BIND_REMOTE_DISPLAY;BIND_ROUTE_PROVIDER;BIND_RUNTIME_PERMISSION_PRESENTER_SERVICE;BIND_SCREENING_SERVICE;BIND_TELECOM_CONNECTION_SERVICE;BIND_TEXT_SERVICE;BIND_TRUST_AGENT;BIND_TV_INPUT;BIND_TV_REMOTE_SERVICE;BIND_VOICE_INTERACTION;BIND_VPN_SERVICE;BIND_VR_LISTENER_SERVICE;BIND_WALLPAPER | The group allows components to BIND to services send requests, receive responses, and communication between them. |
| 10- Broadcast | BROADCAST_CALLLOG_INFO;BROADCAST_NETWORK_PRIVILEGED;BROADCAST_PACKAGE_REMOVED;BROADCAST_PHONE_ACCOUNT_REGISTRATION;BROADCAST_SMS;BROADCAST_STICKY;BROADCAST_WAP_PUSH | The group contains the permissions used to BROADCAST network authorization and removed packages status. |
| 11- Control | CONTROL_INCALL_EXPERIENCE;CONTROL_KEYGUARD;CONTROL_LOCATION_UPDATES;CONTROL_VPN;CONTROL_WIFI_DISPLAY | The group contains the necessary permissions to provide functionality to CONTROL phone calls or to CONTROL critical situations such as that all activities can continue to run even when the phone is locked. |
